# Quantification of Chromosomal Aberrations in Mammalian Cells

**DOI:** 10.21769/BioProtoc.4739

**Published:** 2023-08-20

**Authors:** Inés Paniagua, Jacqueline J. L. Jacobs

**Affiliations:** Division of Oncogenomics, The Netherlands Cancer Institute, Amsterdam, The Netherlands

**Keywords:** Chromosomal instability, Mitosis, DNA repair, DNA replication, Genome integrity, Cancer, Congenital malignancies

## Abstract

Maintenance of genome integrity requires efficient and faithful resolution of DNA breaks and DNA replication obstacles. Dysfunctions in any of the processes orchestrating such resolution can lead to chromosomal instability, which appears as numerical and structural chromosome aberrations. Conventional cytogenetics remains as the golden standard method to detect naturally occurring chromosomal aberrations or those resulting from the treatment with genotoxic drugs. However, the success of cytogenetic studies depends on having high-quality chromosome spreads, which has been proven to be particularly challenging. Moreover, a lack of scoring guidelines and standardized methods for treating cells with genotoxic agents contribute to significant variability amongst different studies. Here, we report a simple and effective method for obtaining well-spread chromosomes from mammalian cells for the analysis of chromosomal aberrations. In this method, cells are (1) arrested in metaphase (when chromosome morphology is clearest), (2) swollen in hypotonic solution, (3) fixed before being dropped onto microscope slides, and (4) stained with DNA dyes to visualize the chromosomes. Metaphase chromosomes are then analyzed using high-resolution microscopy. We also provide examples, representative images, and useful guidelines to facilitate the scoring of the different chromosomal aberrations. This method can be used for the diagnosis of genetic diseases, as well as for cancer studies, by identifying chromosomal defects and providing insight into the cellular processes that influence chromosome integrity.

**Graphical overview**


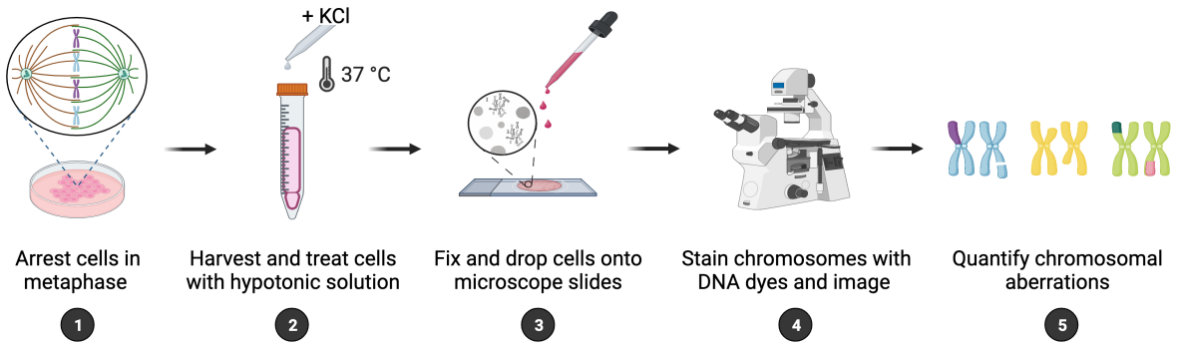


## Background

To maintain a stable genome, cells must accurately duplicate their genetic material before each cell division and ensure efficient signaling and repair of DNA damage. Chromosomal instability (CIN) is a form of genomic instability observed in cancer and many congenital abnormalities, typically associated with numerical or structural chromosome changes (Bakhoum and Cantley, 2018). While numerical CIN is characterized by gain and/or loss of whole chromosomes, structural CIN is characterized by gain, loss, and/or rearrangements of parts of chromosomes. Although significant advances in the detection of CIN have been made with the appearance of quantitative high-throughput imaging cytometry and single-cell genomics, classical cytogenetics still remains as an important method to detect chromosomal aberrations within research and clinical settings (Lepage et al., 2019). Cytogenetic approaches generally involve analyzing metaphase chromosomes stained by DNA dyes such as GIEMSA or DAPI; however, inconsistency in the preparation of chromosome spreads is a major problem. Moreover, the scoring of chromosomal aberrations usually relies on specialized experience, since chromosome spreading artifacts can be easily misinterpreted as genomic changes. Thus, efforts should be made to standardize the assays and refine the analysis of chromosomal aberrations for data interpretation. Here, we report a method that enables fast and reliable preparation of metaphase chromosome spreads from mammalian cells for the purpose of scoring chromosomal aberrations. In brief, cells are treated with a metaphase-arresting substance, harvested, and stained with DNA dyes. Metaphase cells are then analyzed microscopically for the presence of chromosomal aberrations. This method can be used to score gross (i.e., observable with standard staining methods) structural aberrations occurring naturally and following exposure to genotoxic chemicals, such as hydroxyurea, aphidicolin, mitomycin C, etc. While numerical aberrations can also be scored, other methods may be more appropriate for that. In addition, we provide representative images of normal and aberrant metaphases as well as scoring guidelines to facilitate accurate identification and evaluation of chromosomal instability, which is of outmost importance within both research and clinical settings.

## Materials and reagents

1. Gloves and lab coat
2. 10 or 15 cm Petri dish (Greiner or Thermo Fisher Scientific, catalog numbers: 664160 or 168381)
3. 15 and 50 mL screw cap tubes (Sarstedt, catalog numbers: 62554502 and 62547254)
4. Microscope glass slides with a frosted end (Epredia, catalog number: AB00000112EO1MNZ10)
5. Glass coverslips (24 mm × 50 mm) (VWR, catalog number: 631-1574)
6. Paper towel
7. Mammalian cells of interest and appropriate cell culture medium [suggested complete growth medium: Dulbecco’s modified Eagle’s medium (DMEM, Thermo Fisher Scientific, Gibco, catalog number: 41966-029), supplemented with 10% fetal bovine serum (FBS, Capricorn, catalog number: FBS-12A), 100 U/mL penicillin, 100 μg/mL streptomycin (Thermo Fisher Scientific, Gibco, catalog number: 15140-122), and 2 mM L-Glutamine (VWR, catalog number: 392-0441)]
*Note: This protocol is for adherent cells.*
8. Phosphate-buffered saline (PBS) (Thermo Fisher Scientific, Gibco, catalog number: 14190144)
9. Trypsin-EDTA 0.05% (Thermo Fisher Scientific, Gibco, catalog number: 25300-054), store at 4 °C
10. Fetal bovine serum (FBS) (Capricorn Scientific, suggested catalog number: FBS-12A)
11. (Optional) Genotoxic chemical e.g., hydroxyurea (Sigma, catalog number: H8627)
12. Colcemid, KaryoMax colcemid solution, 10 μg/mL (Gibco, catalog number: 15212012), store at 4 °C
13. Potassium chloride (KCl) (Sigma, catalog number: 1049360500)
14. Dimethyl sulfoxide (DMSO) (Sigma, catalog number: 34943)
15. ProLong Gold antifade reagent with DAPI (Thermo Fisher Scientific, catalog number: P36931)
16. Transparent nail polish (any brand)
17. Plastic Pasteur pipette (VWR, catalog number: 612-1684P)
18. Methanol (Honeywell, catalog number: 32213)
19. Glacial acetic acid (Sigma, catalog number: A6283)
20. Hydroxyurea (see Recipes)
21. Hypotonic solution (see Recipes)
22. Fixative solution (see Recipes)

## Equipment

1. Pipettes (Gilson)
2. Vortexer
3. Centrifuge with swing bucket rotor (e.g., Rotina 380, Hettich)
4. Heating block (Stretching Table OTS 40, Medite, catalog number: 401520)
5. Water bath (GFL Shaking Water Baths, catalog number: 1083)
6. Imaging equipment:
  Option 1:
  - Metafer4/MSearch automated metaphase finder system (MetaSystems) equipped with a Zeiss AxioImager Z2 microscope (Carl Zeiss)
  - Objectives: 10×/0.45 and 63×/1.40 oil Plan-ApoChromat
  - Filterset: DAPI (Zeiss Filterset 49, ACR)
  - Camera: CoolCube1
  Option 2:
  - Wide field microscope e.g., Zeiss AxioObserver Z1 microscope
  - Objective: 63×/1.40 oil Plan-ApoChromat
  - Filterset: DAPI (Zeiss Filterset 49, ACR)
  - Camera: ORCA–Flash4.0 V3 Digital CMOS: C13440-20 CU

## Software

1. MetaSystems (version 3.11) software
2. Fiji (version 2.0) software
3. GraphPad Prism (version 9) software

## Procedure

- **Preparation of cultures**
  1. Grow cells in standard culture conditions (e.g., at 37 °C in a humidified atmosphere with 5% CO_2_).
  2. Seed 0.5 × 10^6^ cells into a 10 cm dish 48 h before harvesting, to be ~70% confluent at the time of harvest.
*Notes:*
    - *Adjust the seeding density according to the doubling time of the cell line. You are aiming for your cells to go through approximately 1.5 cell cycles (since many aberrations are lethal or lost during subsequent cell divisions, so they are best observed at the first or second metaphase).*
    - *When working with slow dividing cells, it is better to use a 15 cm dish and seed 1 × 10^6^ cells into it. This will result in a larger cell pellet and a higher percentage of mitotic cells at the time of harvest.*
    - *Since the harvesting of the cells is a delicate process, never harvest more than 6–8 samples at once. When you have more samples, harvest them in several rounds. One harvesting round can take up to 1.5–2 h.*
  3. (Optional) Alternatively, at the time of seeding, treat cells with a genotoxic chemical (e.g., 4 mM hydroxyurea) for 3–6 h, wash two times with PBS, and culture cells in complete growth medium for approximately 1.5 cell cycles.
*Note: When using a 1 M stock of hydroxyurea, add 40 μL of hydroxyurea to 10 mL of medium (for 10 cm dishes).*
- **Arresting cells in metaphase**
  1. On the day of harvest, check whether your cells have the right confluency.
  2. Two hours before harvest, add colcemid directly to the medium to a final concentration of 0.2 μg/mL. When using a 10 μg/mL stock of colcemid, add 200 μL of colcemid to 10 mL of medium (for 10 cm dishes).
*Notes:*
    - *Avoid incubation times longer than 2 h, since prolonged exposure to colcemid results in very compacted chromosomes.*
    - *Volumes indicated in the protocol are for a 10 cm dish; adjust accordingly for larger dishes. For instance, add 400 μL of colcemid to 20 mL of medium when using 15 cm dishes.*
  3. During the colcemid incubation, label 15 mL screw cap tubes for the different conditions, prepare and prewarm the hypotonic solution (see Recipes) at 37 °C, and prepare ice-cold fixative solution (see Recipes) (keep at -20 °C).
  4. After 2 h of colcemid treatment, the effect should be visible: many cells will look rounded, refractile, and appear as if they are about to detach.
- **Harvesting of cells**
*Note: Cells in mitosis round up and lose attachment to the plate. To harvest all mitotic cells (including those floating), save the media and/or the PBS wash as well as the media used to neutralize the trypsin, and collect in a single screw cap tube.*
  1. Remove the media.
*(Optional) Alternatively, save the media in a 15 mL screw cap tube.*
  2. Carefully wash the cells with 5 mL of PBS and recapture them by collecting the PBS in a 15 mL screw cap tube.
  3. Trypsinize and collect the cells in medium containing FBS. Add to the 15 mL tube containing the PBS wash.
*Note: To trypsinize the cells, add 1 mL of trypsin to the dish and incubate for 3–5 min at 37 °C until all cells are loosely floating (this can be seen under a microscope). To neutralize the trypsin, add 7 mL of medium to the dish. Collect cells by resuspending.*
  4. Spin the samples for 5 min at 186× *g* at room temperature (RT).
  5. Remove the supernatant completely and carefully tap to the side of the tube so your pellet will be loose.
  6. GENTLY resuspend your cells in 10 mL of hypotonic solution (prewarmed to 37 °C). Pipette the solution onto the tube wall.
*Note: The addition of KCl swells the cells, so pipette carefully to prevent cell lysis.*
  7. Incubate the cells in the hypotonic solution for 7 min at 37 °C. Slowly invert the tube several times during the incubation to prevent clumping.
*Note: Incubation times may differ per cell line and thus need to be determined empirically. For commonly used cell lines, such as HeLa, MEFs, and RPE1-hTERT, 5–10 min of treatment time is sufficient.*
  8. Spin the cells for 5 min at 186× *g* at RT.
- **Fixation of cells**
  1. Decant the KCl and tap the tube to resuspend the cells in the small volume of KCl that remains (approximately 0.5 mL).
  2. Drop by drop, add 1 mL of ice-cold fixative solution while the cells are slowly and gently being mixed on a vortex.
  3. Fill to 10 mL with the fixative and store at 4 °C overnight or longer; cells can be kept at this stage for months.
- **Metaphase chromosome spread preparation**
*Note: For additional details pertaining to the procedure, please consult Video 1.*
  **Video 1.**
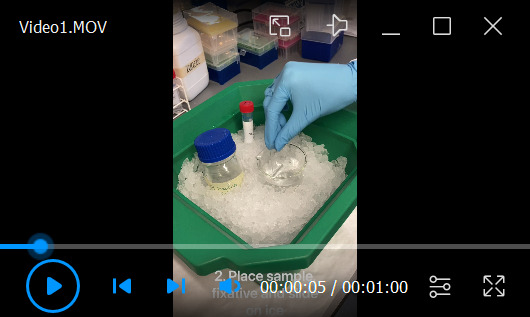
Metaphase chromosome spread preparation. This video shows the procedure to prepare metaphase chromosome spreads, as described in section E, including the fixation and dropping of the cells onto microscope slides.
  1. Spin the cells for 5 min at 186× *g* at RT.
  2. Aspirate the fixative until 0.5–1 mL is left. Resuspend by tapping. Put the tubes on ice.
*Note: Starting with 0.5 × 10^6^ cells seeded in a 10 cm dish that reach 70% confluency at time of harvest yields a cell pellet that, when resuspended in 0.5–1 mL fixative, results in an adequately high cell density for chromosome spread preparation. However, the volume in which you resuspend the cells may be adjusted to change the concentration of the sample and obtain a lower or higher density of metaphases on the slides in subsequent steps.*
  3. Take some (old) pre-cooled fixative solution and put it on ice as well.
  4. Label microscope glass slides for the different conditions (using a pencil since fixative will dissolve the marker). Place the slides in a beaker containing cold water to pre-cool and wet them.
  5. Prepare a humidified 42 °C heating block (place wet paper towels on top of a heating block set to 42 °C).
  6. Place a piece of cloth on the ground to collect splashes. Then, lay down a 50 mL screw cap tube and place a water-wetted slide against it, tilted at a 45° angle.
  7. Using a plastic Pasteur pipette (the tip is wider than a P1000 pipette tip), pipette up and down and take up the cell suspension from step E2. Let the first drops fall into the tube. Then, drop several drops of cell suspension onto different places on a slide whilst standing up. Aim to cover all parts of the slide with each drop but avoid dropping cells on the same spot.
*Note: Slide dropping from a distance (approximately 0.5 m) lets the nuclei fall apart on the slide, so the metaphases will spread. However, make sure that the distance is not too large, since the chromosomes will spread too far apart from each other, which will make it difficult to determine if they are from one or more cells.*
  8. Immediately after dropping, quickly wash by dropping fixative solution across the slide using a plastic Pasteur pipette and place the slides (cell side up) for 3 min on a humidified 42 °C heating block.
*Note: The high temperature and the vapor that is continuously released from the humidified heating block improves the quality of the spreads (Deng et al., 2003).*
  9. Check the slides under a regular light microscope for spreading efficiency. You should see many nuclei and some metaphase chromosomes (Figure 1).
*Note: If you can find 3–5 metaphases in 1 min, there is probably plenty present on the slide. If there are less, you can drop some extra. If too many, dilute sample with fixative and repeat steps E2–E9.*
  10. Air dry the slides in the fume hood for a few hours at RT.
  11. To mount the slides, add a few drops of Prolong Gold antifade with DAPI to the coverslip, pick up the coverslip using the slide, and slowly lay flat. Avoid air bubbles. Gently press the coverslip with a tissue to remove excess mounting medium.
  12. Seal with nail polish.
  13. Slides can be stored up to one week at 4 °C or at -20 °C for longer storage in the dark. The remainder of the sample in the 15 mL tube can be stored in fresh fixative at 4 °C for months.
    **Figure 1.**
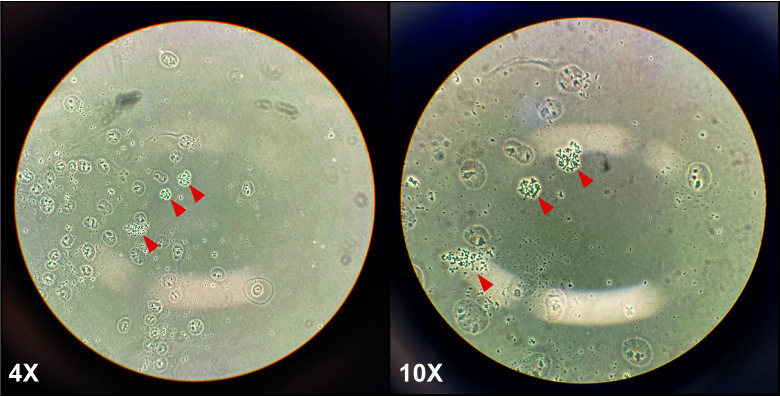
Representative metaphase spreads in a cancer cell line (HeLa) at 4× and 10× magnification. After dropping cells onto microscope slides, many cytoplasm-free nuclei, and some metaphase spreads (red arrows) should be visible. At low magnification (4×, left panel), metaphase chromosomes should look like small black dots; at higher magnification (10×, right panel), the arms of the chromosomes should be visible.

## Data analysis

**Image acquisition and processing**

Digital images of metaphases are captured using the Metafer4/MSearch automated metaphase finder system equipped with an AxioImager Z2 microscope and DAPI filter. In this case, the user reviews the images taken by the automated microscope and selects those metaphases that appear suitable for analysis. This method greatly reduces slide reading time and is thus recommended. Alternatively, metaphases can be manually selected and imaged using a high-resolution microscope, such as a Zeiss AxioObserver Z1 microscope. For this method, though, it is important to establish a systematic way of scanning slides to locate the metaphases (e.g., scanning from side to side or along the length of the slide), to avoid imaging and scoring the same metaphase twice.

At least 50 readable metaphases should be imaged at high magnification (63×, oil immersion). Readable metaphases (i.e., metaphases that are suitable for analysis) are identified by the following criteria:

• Chromosome number within the diploid chromosome number of the working cell line.

• Well-spread chromosomes, with minimal overlap of chromosomes and chromosomes arms.

• Clear and defined chromosome structure, with intact centromeres.

If the required total of 50 metaphases is not obtained, then additional slides should be prepared from the reserved fixed sample.

**Representative images and scoring**

Chromosomal aberrations are quantified from at least 50 metaphase spreads per condition per experiment. Analysis of 50 metaphases per condition and replicate is sufficient to obtain a reliable mean. Data are presented as the average percentage of chromosomal aberrations per metaphase, showing the spread among the replicates of the individual experiments, as previously shown (Paniagua et al., 2022, Figure 3E). Statistical analysis is performed with the appropriate test for multiple comparisons using GraphPad Prism 9.

**Classification**

Chromosomal aberrations are classified into the following categories (Registre, 2016):

• *Chromosome-type* (both sister chromatids affected)

- *Breaks/deletions* (resulting from a DNA break that was not repaired)

- *Exchanges* [resulting from two or more DNA breaks with inappropriate rejoining/repair, within a single chromosome (intrachanges) or between chromosomes (interchanges)]

• *Chromatid-type* (only one sister chromatid affected)

- *Breaks/deletions*

- *Exchanges*

Additionally, other forms of chromosomal aberrations are occasionally observed; these are recorded but not included in the analysis because they do not necessarily involve chromosome breakage.

• Pulverized chromosome/metaphase (total loss of chromosome architectural integrity).

• Gaps (unstained regions on the chromosomes/chromatids, smaller than the width of one chromatid, and with minimal misalignment with the rest of the chromatid/chromosome).

• Polyploidy and/or other forms of aneuploidy.

It is important to be aware that chromatid-type breaks are the most frequently observed aberrations with a sampling time of approximately 1.5 cell cycles from the start of seeding or following drug exposure. After a further cell cycle, some of these chromatid-type aberrations can be converted into chromosome-type aberrations. This is because DNA double-stranded breaks forming and persisting through S-phase will be replicated and become evident as a chromatid-type aberration. If the chromatid-type is not repaired, then it will also be replicated, resulting in the formation of a chromosome-type aberration. For a more comprehensive view of the formation and scoring of chromosomal aberrations, we refer interested readers to an excellent review (Danford, 2012).

**Figure 2.**
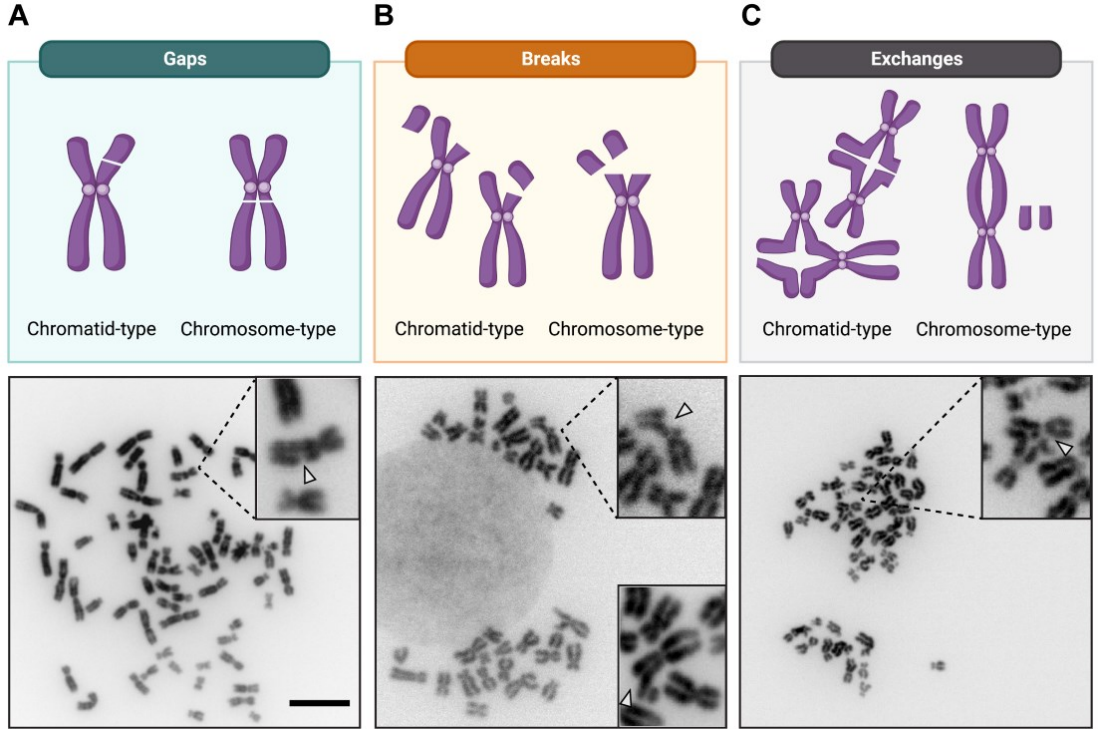
Representative examples of common chromosomal aberrations. Schematic illustration of the three common categories of chromosomal aberrations. Representative metaphase spreads, with normal and aberrant chromosomes in a cancer cell line (HeLa) at 63× magnification, are shown below for each category. A. Gaps: dislocation of the chromatid or chromosome arm with no misalignment. B. Breaks: dislocation of the chromatid or chromosome arm, with misalignment, and an unstained region that is wider than the chromatid width. The insert in the lower right corner is derived from an independent metaphase and shows a different example of how chromosomal breaks may appear. C. (Inter-)exchanges: exchanges involving two chromosomes. Examples of quadriradial figures are shown for the chromatid-type exchanges. Scale bar, 10 μm.

Representative examples of normal and aberrant chromosomes are shown in Figure 2. An example of data quantification is also provided in Table S1.

**Data misinterpretation**

The analysis of chromosomal aberrations has a subjective component; therefore, misinterpretation can occur and result in the (mis-)classification of a normal chromosome as aberrant. Generally, it is better to err on the side of caution and disregard a metaphase altogether if the apparent aberration is unclear. Some common examples for misinterpretations have been highlighted below:

• Crossing-over of sister chromatids can be mistaken for a dicentric chromosome.

• Chromosomes overlapping near centromeres can resemble a chromosome-type exchange (radial).

• Twisted or overlapping chromosomes can be wrongly scored as chromosome rings.

• Secondary constrictions in chromosomes can appear as chromosome gaps.

## Notes

1. Particular attention should be given to standardizing the conditions of swelling, fixation, and slide dropping, so that high quality preparations can be made regularly. Achieving a high mitotic index (total number of metaphases/number of nuclei) is equally important to maximize the sensitivity to genotoxic agents and reduce the slide reading time. To increase the mitotic index at harvest, one can experiment with the cell culture dishes and/or the cell culture density (confluency should always be avoided for cells growing in monolayers).
2. In cell lines with high levels of chromosomal instability and in some cancer cell lines, a basal level of chromosomal aberrations can be detected.
3. Certain genotoxic treatments, including hydroxyurea, have the potential to alter the chromosomal copy number of the treated cells. Thus, it is recommended to control for copy number variation (CNV) by counting the total number of chromosomes per metaphase. In case the manipulation results in CNV, the quantification of chromosomal aberrations should then be plotted as chromosomal aberrations per total number of chromosomes (in contrast to the standard plotting of chromosomal aberrations per metaphase).

## Recipes

1. **Hydroxyurea**
  1 M in DMSO
  Dissolve 0.38025 g of hydroxyurea in 5 mL of DMSO
  Aliquot in small volumes (0.5 mL) and store at -20 °C for up to four months
2. **Hypotonic solution**
  0.075 M KCl in dH_2_O
  Prewarm the required amount of 0.075 M KCl in a 37 °C water bath before each use
3. **Fixative solution**
  Three parts methanol
  One part glacial acetic acid
  Make fresh each time
  Use ice-cold

## Supplementary information

The following supporting information can be downloaded here:

1. Table S1. Example data quantification
